# Housing Arrangement and Location Determine the Likelihood of Housing Loss Due to Wildfire

**DOI:** 10.1371/journal.pone.0033954

**Published:** 2012-03-28

**Authors:** Alexandra D. Syphard, Jon E. Keeley, Avi Bar Massada, Teresa J. Brennan, Volker C. Radeloff

**Affiliations:** 1 Conservation Biology Institute, La Mesa, California, United States of America; 2 United States Geological Survey, Western Ecological Research Center, Sequoia-Kings Canyon Field Station, Three Rivers, California, United States of America; 3 Department of Ecology and Evolutionary Biology, University of California Los Angeles, Los Angeles, California, United States of America; 4 Department of Forest and Wildlife Ecology, University of Wisconsin-Madison, Madison, Wisconsin, United States of America; University of Bristol, United Kingdom

## Abstract

Surging wildfires across the globe are contributing to escalating residential losses and have major social, economic, and ecological consequences. The highest losses in the U.S. occur in southern California, where nearly 1000 homes per year have been destroyed by wildfires since 2000. Wildfire risk reduction efforts focus primarily on fuel reduction and, to a lesser degree, on house characteristics and homeowner responsibility. However, the extent to which land use planning could alleviate wildfire risk has been largely missing from the debate despite large numbers of homes being placed in the most hazardous parts of the landscape. Our goal was to examine how housing location and arrangement affects the likelihood that a home will be lost when a wildfire occurs. We developed an extensive geographic dataset of structure locations, including more than 5500 structures that were destroyed or damaged by wildfire since 2001, and identified the main contributors to property loss in two extensive, fire-prone regions in southern California. The arrangement and location of structures strongly affected their susceptibility to wildfire, with property loss most likely at low to intermediate structure densities and in areas with a history of frequent fire. Rates of structure loss were higher when structures were surrounded by wildland vegetation, but were generally higher in herbaceous fuel types than in higher fuel-volume woody types. Empirically based maps developed using housing pattern and location performed better in distinguishing hazardous from non-hazardous areas than maps based on fuel distribution. The strong importance of housing arrangement and location indicate that land use planning may be a critical tool for reducing fire risk, but it will require reliable delineations of the most hazardous locations.

## Introduction

As the frequency, extent, and severity of wildfires are surging across the world [1], [2], so too are the ecological, social, and economic consequences. Residential losses associated with wildland fire have escalated globally [3]–[5], and recent fire events have resulted in billions of dollars of damage per event [6]. The problem is particularly critical in Mediterranean-climate regions of the world, where major metropolitan centers are juxtaposed with highly flammable ecosystems [7]. Since the 1950s, southern California has experienced the highest losses in property and life in the U.S., averaging 500 homes per year [8]. Here we show that the arrangement and location of structures strongly affects their susceptibility to being destroyed in a wildfire, and that empirically based maps developed using housing density and location can better identify hazardous locations than fuel-based maps.

The escalation of wildland fire losses is typically attributed to housing development within or adjacent to wildland vegetation (i.e., the “wildland-urban interface”) [6], [9], changing climate conditions [1], or an accumulation of hazardous wildland fuels [10]. The primary preventive strategy used for reducing fire impacts has been the manipulation of wildland vegetation to reduce hazardous fuels. The U.S. federal government has strongly promoted and funded fuel reduction treatments to mitigate fire hazard, and federal land management agencies spent billions of dollars (e.g., $2.7 billion from 2001–2006) to treat millions of hectares within the last decade [10]. Yet, while costs for suppression and treatment have nearly tripled since 1996 [11], the fire problem has only gotten worse.

With the growing realization that wildland fuel manipulations can alter fire outcomes only to a limited extent, the need for alternatives has risen. For example, a structure's survival during a wildfire depends largely on its building materials and the characteristics of fuels in its immediate surroundings [3], suggesting that fire hazard can be reduced by homeowner actions to protect the structure [12].

However, what remains unclear is to what extent property loss depends on the role of land planning and the placement and arrangement of homes relative to the spatial patterns of wildland fire hazards. Past land-use decision-making has allowed homes to be constructed in highly flammable areas, and this may be one of the roots of the fire problem [13]. Although it is not feasible to change current housing patterns, homes in the most hazardous locations could be identified and prioritized for fire protection efforts, and land use planning and regulation may potentially be a powerful tool for reducing future property loss [14], especially in areas such as southern California where substantial future housing growth is expected [15], and across the western US, where further development is expected in a substantial proportion of the wildland-urban interface [16].

If land use regulation and planning are to effectively reduce wildland fire loss, they have to be based on solid understanding of what landscape factors most significantly contribute to wildfire danger and where to locate and arrange homes to reduce fire hazard. Currently, most fire hazard maps are based on expert knowledge of how fuel and fire history determine threats to a given community e.g., [17]–[19]. Similar fire hazard maps have been created for the state of California that identify communities at risk and areas of substantial fire threat to people. These maps are readily available [20] and widely used. Fire hazard maps, however, are only effective if they accurately delineate areas where property loss is most likely to occur. Whether this is the case or not is unknown since most have never been evaluated against empirical data.

**Figure 1:**
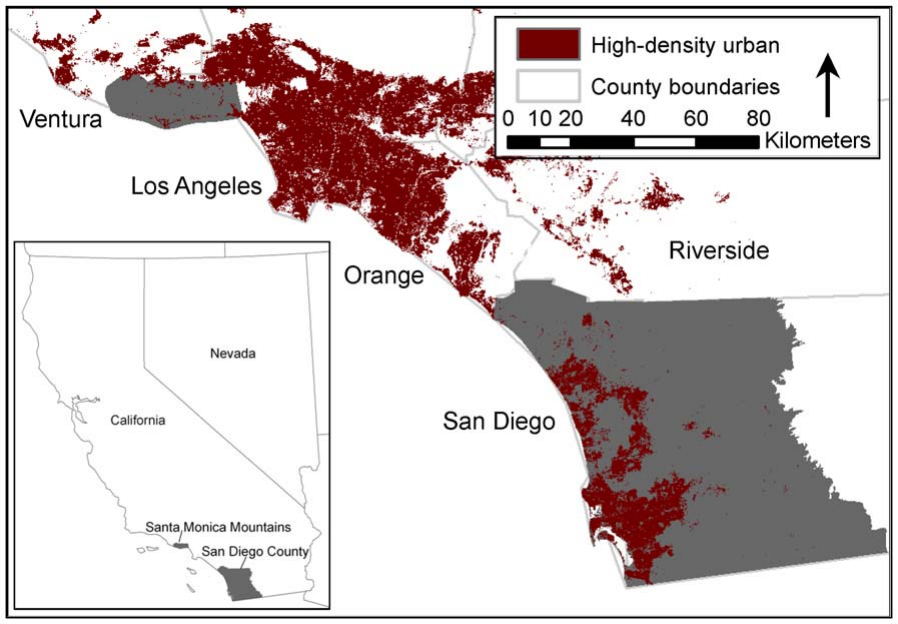
The Santa Monica Mountains and San Diego County, California, USA. Study areas in gray. The Santa Monica Mountains are located in Ventura and Los Angeles counties, and both study areas are located within the South Coast Ecoregion of California, USA. Study areas in gray.

We constructed a complete database of structure locations in two extensive, fire-prone regions of southern California and identified which structures were destroyed or damaged by wildfires since 2001 (Fig. 1). These two regions were the Santa Monica Mountains, one of the largest wildland open space areas adjacent to the Los Angeles metropolitan area and San Diego County, site of major wildfire losses in both 2003 and 2007 [20]. Based on these data, we used logistic regression and maximum entropy analysis to answer three questions: 1) What is the relative importance of housing arrangement (i.e., the spatial pattern of residential structures), location, and environment in explaining property loss from fire? 2) How well do currently available statewide fuel-based maps of fire hazard correspond to actual wildfire impacts? 3) Can fire hazard maps based on empirical data and an expanded set of explanatory variables successfully predict local-scale housing losses?

## Results

In the Santa Monica Mountains, 3% of 36,399 structures were located within the boundaries of 10 large fires that occurred from 2001 to 2009. In these fires, 173 homes, guest houses, or outbuildings were destroyed and an additional 140 were damaged. For the second study region in San Diego County, 4% of 687,869 structures were located within one of 40 fire perimeters. In these fires, 4315 structures were completely destroyed and an additional 935 were damaged.

**Figure 2:**
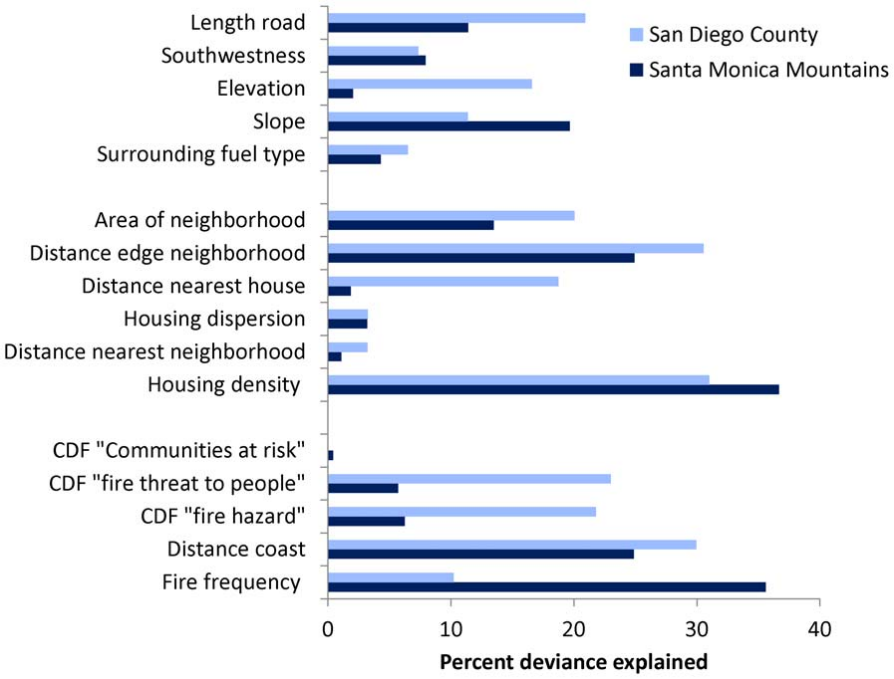
Percent deviance explained for generalized additive models (GAMs). GAMs explain the influence of firefighter access, biophysical variables, structure arrangement, and structure location on burned structures from fires during 2001–2010 in the Santa Monica Mountains, CA and San Diego County, CA. CDF – California Department of Forestry and Fire Protection.

**Figure 3:**
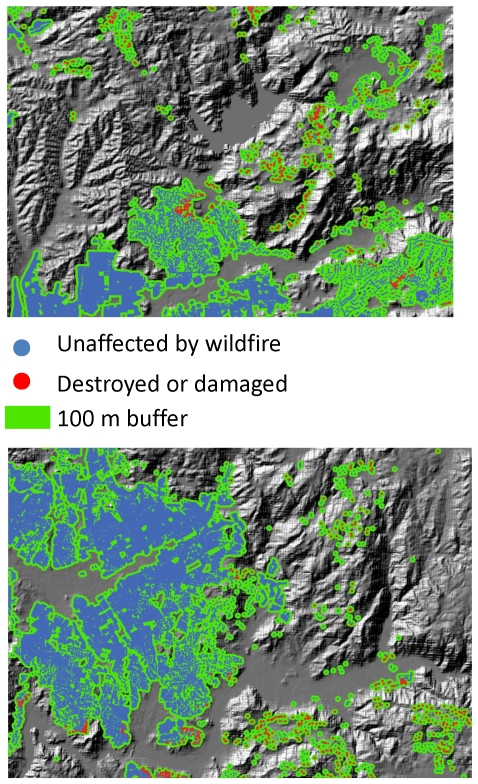
Maps from portions of San Diego County illustrating how housing arrangement influences the likelihood that a house will be lost from wildfire. Structures most likely to be burned by fires (in red) were: in areas with low to intermediate structure density; in small, dispersed housing clusters, close to the edge of the housing cluster, at intermediate distance to the nearest structure or housing cluster than structures that were unaffected (in blue).

In both study regions, the spatial arrangement of structures (Table 1) significantly influenced the likelihood of property loss (i.e., destruction or damage) (Figs. 2 and 3). Property loss was more likely in smaller, more isolated housing clusters with low- to intermediate housing density and fewer roads, although road density was insignificant after accounting for spatial autocorrelation in the Santa Monica Mountains (Table 2). Structures located near the edges of developments, or in housing clusters on steep slopes, were also more susceptible. Many relationships were nonlinear, with the highest property loss occurring when structures were at intermediate distances to other structures or housing clusters.

**Table 1:** Variables analyzed for explaining structure loss in the Santa Monica Mountains and San Diego County.

*Variable*	*Source*	*Description*
Fire frequency 2001	CDF* Fire perimeter overlays	Number of fires (2001–2010)
Distance to coast	Derived from coastline of county	Continuous distance in meters
Fire threat	CDF*	Ranking from 1 to 5
Fire threat to people	CDF*	Ranking from 1 to 5
Communities at risk	CDF*	Binary, at risk or not at risk
Housing density	Derived from digitized structures	Structures per hectare
Distance nearest housing cluster	Derived from 100 m buffer of structures	Continuous distance in meters
Housing dispersion	Derived from 100 m buffer of structures	Standard deviation/mean distance between structures in housing cluster
Distance to nearest structure	Derived from digitized structures	Continuous distance in meters
Distance to edge of housing cluster	Derived from digitized structures	Continuous distance in meters
Area of housing cluster	Derived from 100 m buffer of structures	Squared meters
Elevation	US Geological Survey digital elevation model (DEM	30 meters
Slope	Derived from the DEM	Percent slope
Southwestness	Derived from the DEM	SW = con(aspect(<dem>) = = −12, 201,(cos(((aspect(<dem>)−255) div deg)+1) * 100)))
Road length	US Census Bureau TIGER/Line files	Meters

*California Department of Forestry Fire and Resource Assessment Program.

**Table 2:** Model coefficients for generalized linear models (GLMs) estimated with and without autocovariate terms in the Santa Monica Mountains and San Diego County.

	*Linear*	*Autocovariate linear*	*Quadratic*	*Autocovariate quadratic*	*P-value*
*Santa Monica Mountains*					
Fire frequency 2001	0.860	0.440			<0.001
Distance coast	0.004	0.002	−7.0E-07	−4.0E-07	<0.001
CDF Fire threat	5.900	2.880	−8.5E-01	−3.9E-01	<0.001
CDF Fire threat people	3.070	1.540			<0.01
CDF Communities risk	−0.540	−0.280			NS
Housing density	1.010	1.130	−3.9E-01	−4.0E-01	<0.001
Distance housing cluster	0.006	0.004	−1.0E-05	−7.0E-06	NS
Housing dispersion	2.280	2.670			<0.001
Distance structure	0.020	0.020	−3.0E-05	−2.0E-05	<0.001
Distance edge	−0.021	−0.017			<0.001
Area housing cluster	−2.0E-07	−8.0E-08			<0.001
Slope	0.033	0.016			<0.001
Elevation	−0.001	−0.001			0.01
Southwestness	−0.002	0.002			NS
Road length	−2.0E-05	−2.0E-05			NS
*San Diego County*					
Fire frequency 2001	1.53	1.05	−0.33	−0.22	<0.001
Distance to coast	3.0E-04	3.0E-09	2.0E-04	2.0E-09	<0.001
CDF Fire threat	−0.54	−0.68	0.189	0.17	<0.001
CDF Fire threat people	2.27	1.69			<0.001
CDF Communities risk	−0.93	−0.51			<0.001
Housing density	−0.99	−0.47			<0.001
Distance housing cluster	0.005	0.004	−4.0E-06	−1.0E-06	<0.001
Housing dispersion	−3.08	−1.68	0.865	0.542	<0.001
Distance structure	0.007	0.004	−5.0E-06	−2.0E-06	<0.001
Distance edge	−0.02	−0.01			<0.001
Area of housing cluster	−2.0E-08	−7.0E-09			<0.001
Slope	0.17	0.12			<0.001
Elevation	0.001	0.003			<0.001
Southwestness	−0.005	−0.003			NS
Road length	−1.0E-06	−7.0E-07			<0.001

Quadratic terms were evaluated for all models, and coefficients are only provided for those models in which the quadratic term was significant in the non-spatial model.

In addition to spatial arrangement, a structure's location on the landscape was also a highly significant predictor of property loss (Fig. 2). In both study regions, property loss was significantly related to a structure's distance from the coastline, but the relative effect varied. In the Santa Monica Mountains, property loss occurred disproportionately closer to the coast, whereas structures farther from the coast were most susceptible in San Diego County (Tables 2 and 3).

The other significant location-dependent variable affecting property loss was historical fire frequency (Fig. 2). In the Santa Monica Mountains, this was the single most important predictive variable. Here, property loss was most likely in areas of historical high fire frequency, which corresponded with wind corridors. Fire frequency was also a significant variable in San Diego County, but here the relationship was nonlinear.

**Figure 4:**
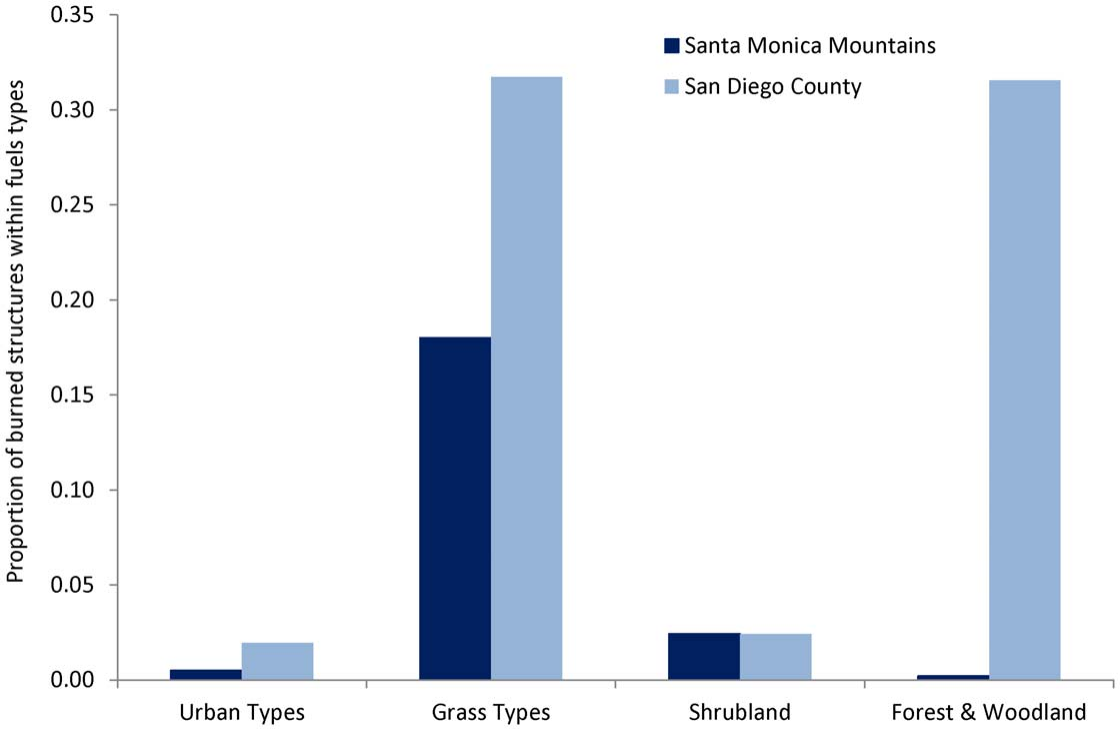
Proportion of burned structures within broad fuels types in the Santa Monica Mountains and San Diego County.

Property loss was more likely to occur when structures were surrounded by wildland vegetation rather than by urban or impervious areas (Fig. 4). However, property loss was also more (Santa Monica Mountains) or as likely (San Diego County) to occur within herbaceous fuel types than within the higher fuel-volume woody types that are typically considered as the most hazardous fuels.

Variables with correlation coefficients greater or equal to 0.7 in the Santa Monica Mountains included road length and area of housing cluster (0.95) and elevation and distance to coast (0.72). In San Diego County, pairs of correlated variables also included road length and area of housing cluster (0.99), distance to nearest structure and distance to nearest housing cluster (0.71). Distance to coast was correlated with housing density (−.71) and elevation (0.89). To develop multiple-regression models, we removed elevation and road length from consideration in the Santa Monica Mountains, because they explained less variation than the variable with which they were correlated. For the San Diego County analyses, we removed distance to coast, road length, and distance to nearest housing cluster.

The multiple-regression GAM model for the Santa Monica Mountains included fire frequency, housing density, distance to edge of housing cluster, distance to coast, slope, area of housing cluster, southwestness, fuel type, housing dispersion, distance to nearest structure and housing cluster. Only nonparametric terms were selected, except fuel type, which was categorical. The deviance explained for the model was 65.7%, and the area under the curve (AUC) of receiver operating characteristic (ROC) plots, indicating the ability of the model to discriminate between burned and unburned structures on test data (20%), was 0.82.

The multiple-regression GAM model for San Diego County included housing density, distance to edge of housing cluster, area of housing cluster, elevation, fire frequency, fuel type, and housing dispersion. All terms included in the model were nonparametric except for distance to edge of neighborhood, which was linear, and fuel type. The deviance explained for the model was 45.5%, and the AUC was 0.87.

**Figure 5:**
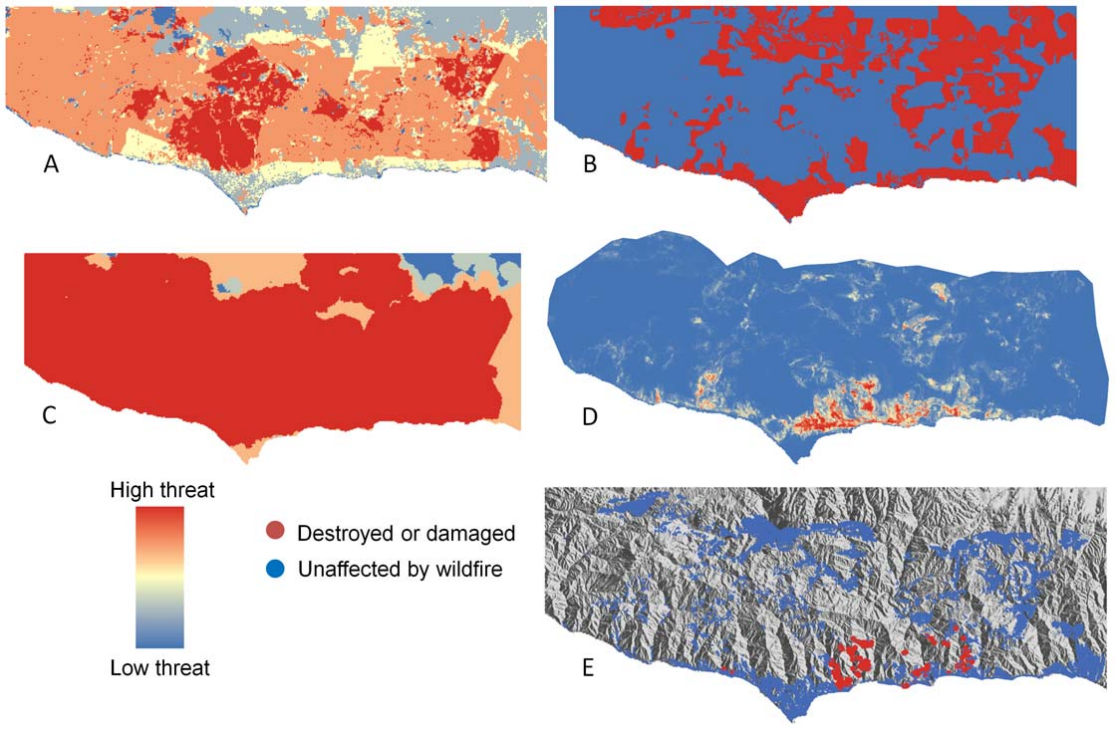
Fire hazard maps versus actual burned structures in the Santa Monica Mountains. (A) CDF “Fire threat” (B) CDF “Communities at risk” (C) CDF “Fire threat to people (D) Empirically based map showing probability of structure being burned by fire (E) Structures that were destroyed or damaged (red) and unaffected (blue) by wildfire from 2001–2010. CDF – California Department of Forestry and Fire Protection.

**Figure 6:**
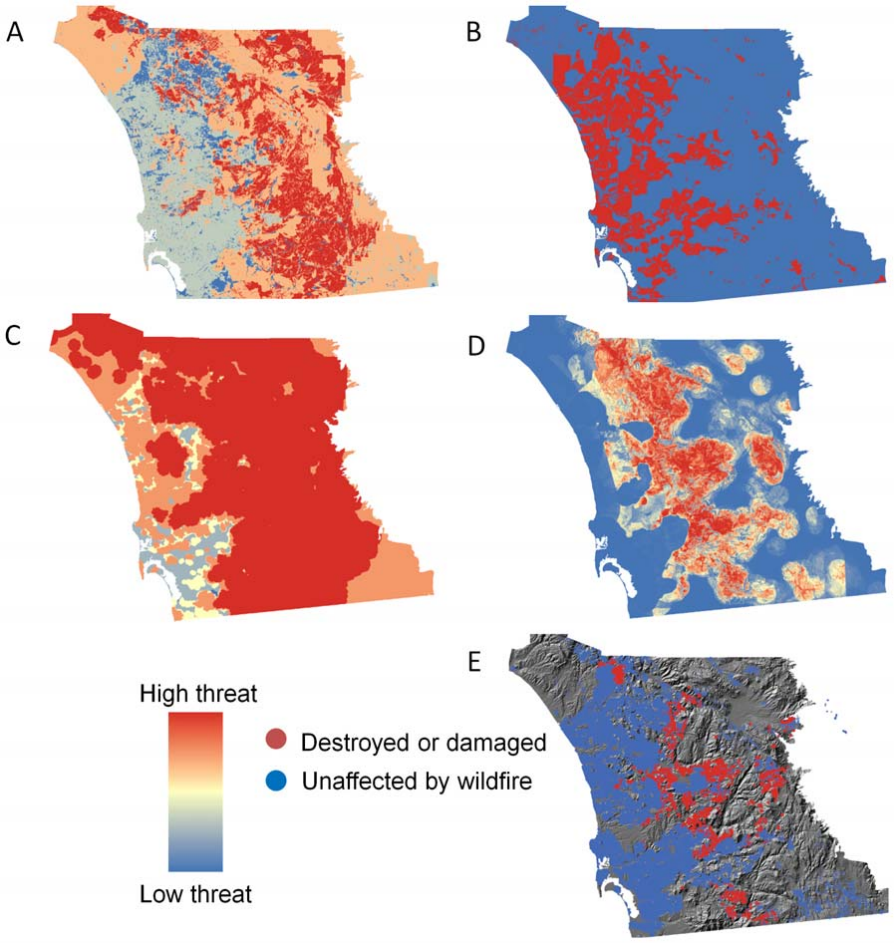
Fire hazard maps versus actual burned structures in San Diego County. (A) CDF “Fire threat” (B) CDF “Communities at risk” (C) CDF “Fire threat to people (D) Empirically based map showing probability of structure being burned by fire (E) Structures that were destroyed or damaged (red) and unaffected (blue) by wildfire from 2001–2010. CDF – California Department of Forestry and Fire Protection.

Our fire-hazard maps developed with the Maxent model using empirical data and multiple explanatory variables (Figs. 5 and 6) performed well. The AUC of receiver operating characteristic (ROC) plots on test data (15% withheld) was 0.987 for the Santa Monica Mountains and 0.923 for San Diego County.

**Figure 7:**
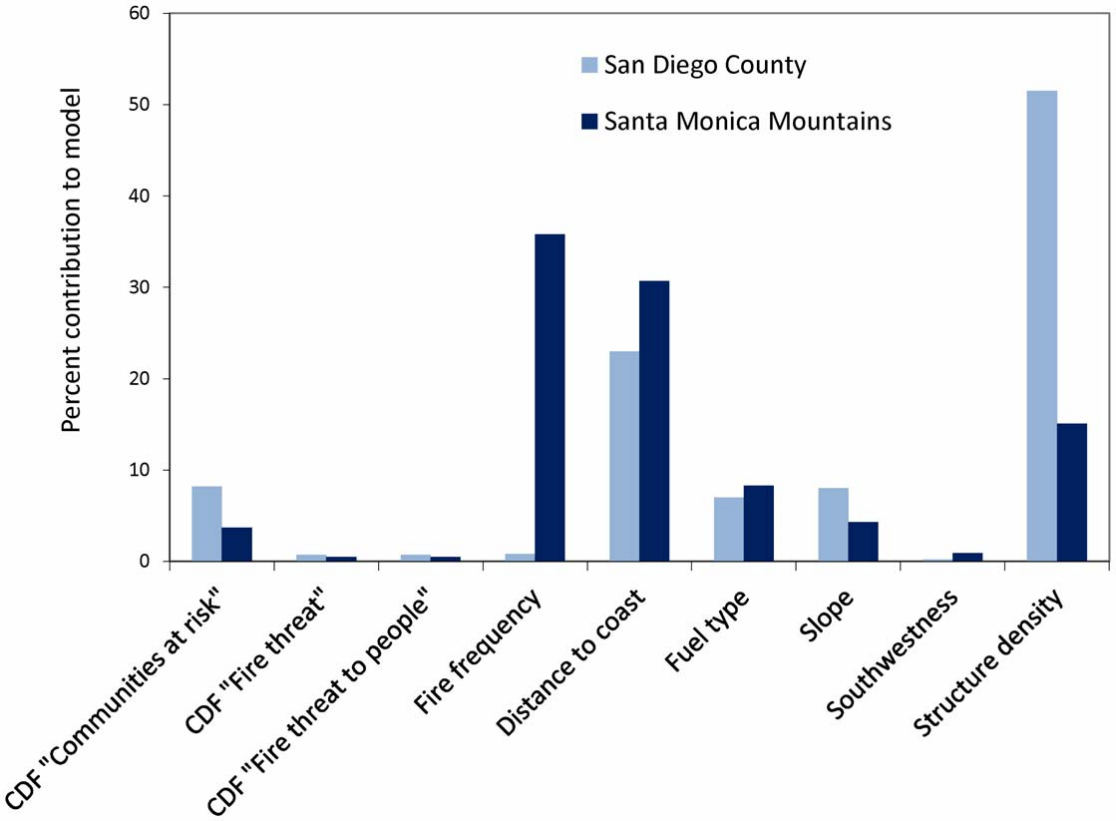
The percent contribution of explanatory variables in Maxent empirical fire hazard model. CDF – California Department of Forestry and Fire Protection.

**Figure 8:**
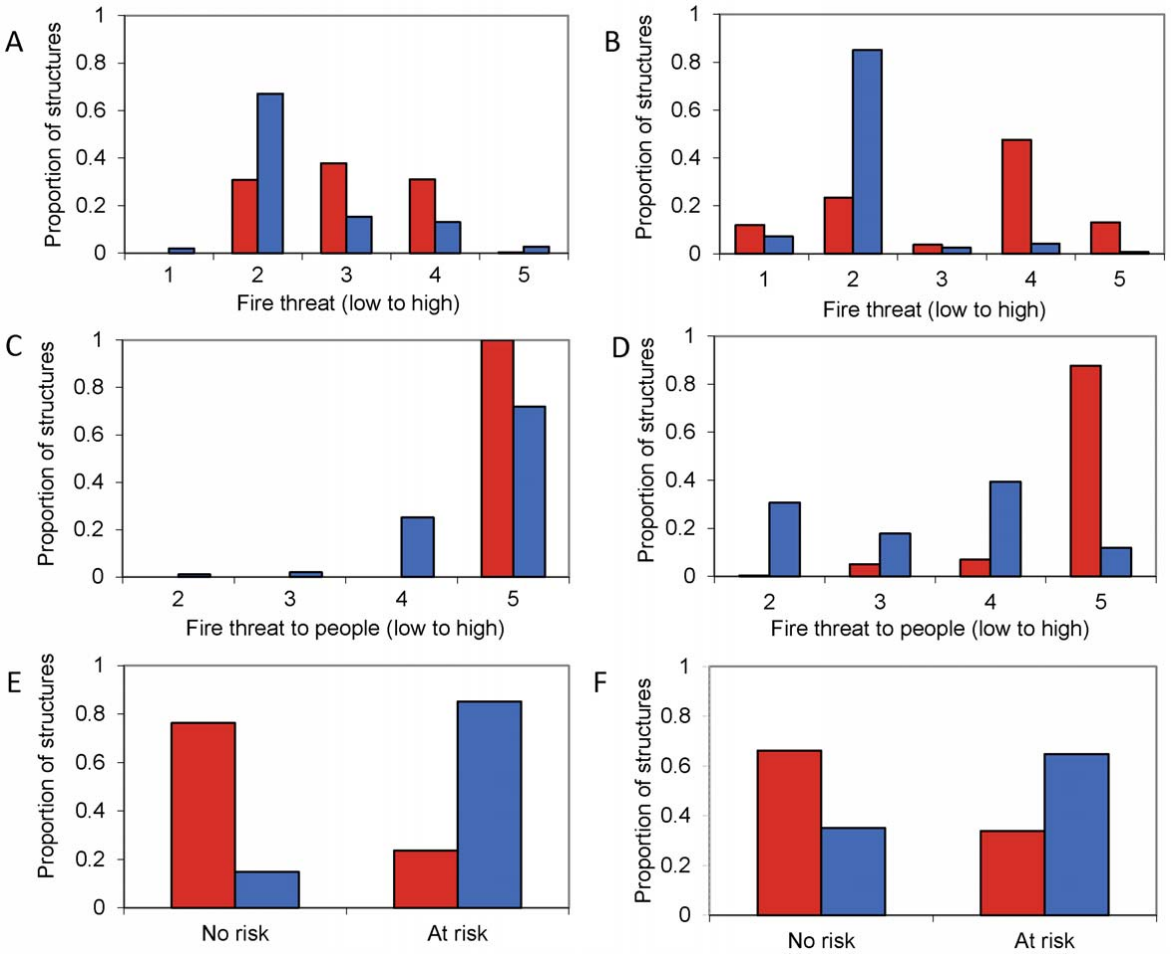
Distribution of actual burned structures in classes of statewide fire hazard maps. Proportion of structures burned (in red) or unaffected (in blue) distributed within map classes of: (A) CDF “Fire threat” in Santa Monica Mountains. (B) CDF “Fire threa” in San Diego County. (C) CDF “Fire threat to people” in Santa Monica Mountains (D) CDF “Fire threat to people” in San Diego County (E) CDF “Communities at risk” in Santa Monica Mountains (F) CDF “Communities at risk” in San Diego County. CDF – California Department of Forestry and Fire Protection.

In contrast, statewide fire-hazard maps developed using fuel rank and fire rotation were unable to predict which structures were burned by fire (Fig. 7). This poor performance of the statewide maps was also evident through visual comparison with maps of actual property loss (Figs. 5 and 6). Similarly, property loss was not substantially higher in the highest hazard or communities-at-risk areas of the statewide maps. In most cases, property loss was evenly divided among hazard levels (Fig. 8A and 8B), and even where a substantial proportion of burned structures were located in areas mapped as high fire hazard, most of the unaffected structures were also distributed in these high-hazard areas, suggesting high commission error (Fig. 8C and 8D). The most worrisome finding was that the majority of property loss occurred in areas not designated as at-risk (Fig. 8E and 8F).

The results of all sensitivity analyses indicated that the results were robust: the importance and ranking of variables remained essentially the same for all data sets at different buffer distances and certainty classifications (Table 3). Differences in results were slightly larger using different buffer distances than using all burned structures across a range of certainty levels versus all destroyed structures classified at the highest level of certainty. The main difference between the 200 and 100-m buffer analysis was that housing density was somewhat less important while distance to nearest housing cluster and southwestness were somewhat more important using the 200-m buffer in the Santa Monica Mountains. In San Diego County, housing dispersion and distance to the edge of housing cluster were somewhat more important using the 200-m buffer. We also found no substantial difference in results for the Maxent models.

**Table 3:** Percent deviance explained in generalized additive models (GAMs) for structures that were destroyed or damaged (Burned) and destroyed with the highest certainty (Destroyed); and for burned structures analyzed using a 200 m buffer distance (200 m).

	*Burned*	*Destroyed*	*200 m*	*Relationship*
*Santa Monica Mountains*				
Fire frequency 2001	35.59	31.63	NA	Positive
Distance coast	24.86	22.85	NA	Intermediate
CDF fire threat	6.23	4.37	NA	Intermediate
CDF fire threat people	5.69	5.01	NA	Positive
CDF Communities at risk	0.42	0.81	NA	Negative
Housing density	36.68	33.19	14.04	Intermediate
Distance housing cluster	1.08	1.46	14.23	Intermediate
Housing dispersion	3.18	2.23	4.24	Positive
Distance structure	1.85	2.17	NA	Intermediate
Distance edge	24.92	33	16	Negative
Area of housing cluster	13.47	12.88	18.06	Negative
Surrounding fuel type	4.3	3.18	NA	NA
Slope	19.66	17.79	18.31	Positive
Elevation	2.04	0.78	1.62	Negative
Southwestness	7.93	8.91	16.1	NA
Road length	11.4	11.2	13.98	Negative
San Diego County				
Fire frequency 2001	10.2	10.6	NA	Intermediate
Distance coast	30.0	28.19	NA	Intermediate
CDF fire threat	21.8	20.4	NA	Intermediate
CDF fire threat to people	23.9	24.1	NA	Positive
CDF Communities at risk	0.0	0.02	NA	Negative
Housing density	31.0	28.16	21.59	Negative
Distance housing cluster	3.2	2.92	0.97	Intermediate
Housing dispersion	3.3	2.85	8.62	Parabolic
Distance structure	18.7	15.73	NA	Intermediate
Distance edge	30.5	28.74	54.76	Negative
Area of housing cluster	20.1	16.41	10.63	Negative
Surrounding fuel type	6.5	4.90	NA	NA
Slope	11.4	13.94	10.61	Positive
Elevation	16.6	25.5	19.75	Positive
Southwestness	7.3	6.98	4.17	NA
Road length	20.9	19.6	15.4	Negative

The buffer distance used in all other analysis was 100 m. Relationship describes the shape of the response curve for all models. Ïntermediate signifies a nonlinear relationship in which values were highest at intermediate levels of the variable. Values listed as NA in 200 m were for variables that were only analyzed at the level of the individual house.

After adding a spatial term, spatial autocorrelation was no longer present in the residuals of any of the models (Table 2). Also, although there were small differences in the coefficients between spatial and non-spatial models, the direction of influence consistently remained the same. The only variables that were no longer significant after accounting for spatial autocorrelation included the CDF communities at risk map, the distance to the nearest housing cluster, southwestness, and road length for the Santa Monica Mountains, and southwestness for San Diego County.

## Discussion

Wildfire is a key process that interacts with all major components of the earth system, but fire frequency, extent, and/or severity are on the rise [1], [2], [21], [22]. Residential losses to wildfire have also escalated despite enormous investments in wildland fuel manipulation, improvements in fire-safe codes and building regulations, and advanced fire suppression tactics. Therefore, our finding that housing arrangement and location were the most important contributors to property loss supports the notion that patterns of land use may be partly responsible for property loss in the wildland-urban interface [13].

One reason that property loss is related to the arrangement of housing across the landscape may be that the amount and arrangement of human infrastructure also strongly and nonlinearly influence wildfire ignitions and frequency [7], [23], [24]. Therefore, the places where homes are most likely to burn may also be the places where fires are most likely occur, which is partly a function of the distribution of people. Thus, there may be spatial interactions and feedbacks between fire and housing patterns.

In southern California, as in many regions, humans cause most fires [7], [23]–[25]. Thus, population growth and housing development increase fire frequency. Yet, although urban expansion increases fire frequency in general, the highest hazard tends to be in low-density housing areas, where structures are interspersed with wildland vegetation [9]. Scattered, isolated structures are more difficult for firefighters to defend, and poor firefighter access may explain why housing clusters with fewer roads were more vulnerable in San Diego County. However, there can also be situations in which high housing density contributes to structure-to-structure fire spread e.g., [26], depending on their flammability [27].

The importance of a structure's location on the landscape relative to the coast and historical patterns of fire frequency shows that certain places are more fire-prone than others, which in turn reflects how biophysical and human variables together create conditions that are particularly conducive to wildfire occurrence [2]. In our study areas, these relationships are also likely a function of a structure's location relative to predominant wind patterns and direction [28]. In the Santa Monica Mountains, certain fire corridors tend to burn repeatedly, and winds funnel down these corridors toward vulnerable structures located directly in their path. Here, the high-density coastal strip is narrow, and homes are closer to continuous vegetation than in San Diego County, where high-density development extends inland for much greater distances. This may be why houses were more likely to burn at a closer distance to the coast in the Santa Monica Mountains than in San Diego County. The low-density, high-risk areas in San Diego County are located farther inland where, if an ignition occurs there under extreme wind conditions, the fire is in its initial stages. Santa Ana winds blow from west toward the coast, and they are particularly dangerous in the beginning because they are usually most explosive and fast-moving right after they start, and it takes time to mobilize firefighting resources. Thus, the significance of distance to coast may be a proxy for other variables, such as the juxtaposition of housing density, contiguous fuels, and location relative to predominant wind patterns.

The importance of historical fire frequency suggests that, at least in non-forested ecosystems, fuel age may not be an important predictor of home loss [25], despite the fact that fuel age and time-since-fire maps are often used to delineate fire hazard. In fact, substantial property loss occurred when the primary surrounding fuel type was low fuel-volume grasslands. Although this result may seem counter-intuitive, herbaceous fuels tend to have low fuel moisture, facilitate high wind speeds and fire spread, and have low heat requirements for ignition, thus promoting longer fire seasons and high fire frequency [29], [30]. Grasslands also tend to ignite quickly, then carry fires into shrublands or woodlands [31]. These results suggest a need to reexamine the assumptions used in existing hazard maps and the management practice of converting shrublands to grasslands.

Fire hazard in the CDF statewide maps, as with most hazard maps [17]–[19], [32], depends largely on the assumption that fuel properties are the primary contributors to fire danger. However, our empirical data indicate that, at least at the local scale considered here, fuel was not as significant as measurable factors related to the arrangement and location of structures. This is likely because the influence of fuel is complex and interacts with other risk factors [33]. Therefore, our empirical maps developed using a more comprehensive set of predictor variables, including fuel type, housing arrangement and location, and other environmental variables, performed better in distinguishing hazardous from non-hazardous areas.

Another reason for the discrepancy in map performance may be related to differences in mapping approach: while our approach used empirical data on actual structure loss, the statewide maps were developed based on a priori assumptions of where hazard is expected to be highest. At larger scales, such as the state level, the CDF fuel-based maps would likely perform better at picking out where homes are most vulnerable to fires. We also did not evaluate the CDF maps developed for local responsibility areas, which may better capture finer-scale patterns of hazard in local jurisdictions.

The fact that unburned structures in our analysis were more likely to be located in “communities at risk,” whereas burned structures were more likely to be located outside of high-risk areas is potentially due to two reasons. At the most basic level, this may simply be caused by an incorrect identification of communities at risk. However, we caution that the discrepancy may also be due to scale effects and the definition of “community at risk.” At a broad scale, “communities at risk” are likely located within areas that generally have the potential for hazardous fires, and places with more houses in such a danger zone are more likely to be identified as a “community at risk.” However, at the structure level, low-housing density significantly increases the chance a house will burn – while it decreases the likelihood that at home will be included in a “community at risk.” In summary, our results support the notion that property loss is a function of many physical and biological factors, in addition to characteristics of home construction and maintenance that we did not consider, such as roofing, construction materials, and home landscaping.

The effects of housing arrangement and location on the likelihood that a house will be destroyed or damaged by wildfire suggest that land use planning may be a critical tool for reducing fire hazard. Restricting development from hazardous locations has been effective for other hazards, such as flooding and the prevention of building on floodplains [34]. In the case of fire, new structures should be located and arranged in ways that not only minimize their exposure to hazard, but may also limit the increase in fire occurrence that often accompanies urban development. For example, our results suggest that in both study areas, new development would have a lower likelihood of burning if it were located away from fire-prone areas, such as wind corridors or steep slopes, and if new structures were arranged in intermediate-to high-density neighborhoods designed to minimize the amount of interface between homes and wildland vegetation. New development within large, existing urban areas, which typically also have better firefighter access, would also lower the likelihood of burning, compared to new development in more isolated, remote settings. Land use planning that considers minimizing future structure loss and prioritizing other fire prevention actions would be more informed with maps that reliably differentiate the most hazardous locations than with maps currently used for this purpose. Although the direction of influence was the same for most variables in the two study regions, the relative importance varied, and the distance from coast and elevation had opposite effects. This supports the notion that hazard is place-specific [35], and fire hazard mapping should therefore be individualized for specific landscapes.

## Materials and Methods

### Data and digitizing structures

We explained property loss by comparing structures that were burned (i.e., destroyed or damaged) by wildfires to those structures that were unaffected. The likelihood of a house burning in a fire has two major components: the first is the likelihood that there will be a fire, and the second is the likelihood that a structure will burn if there is a fire. That ‘total’ likelihood required us to include both structures inside and outside of fire perimeters in the model. We also wanted to account for the full range of variation for the explanatory variables because planning decisions occur at a landscape scale, not just for a subset of structures within fire perimeters. Therefore, we digitized and analyzed all residential structures within the Santa Monica Mountains National Recreation Area in Ventura and Los Angeles counties, California as well as the portion of San Diego County that falls within the South Coast Ecoregion. Using onscreen digitizing, we carefully scanned the most recent aerial imagery available in Google Earth for each study area and placed a point over every visible structure. We digitized all structures, including homes, outbuildings, and guest houses, because we assumed that the factors explaining which homes burned were similar to those explaining the burning of other structures. Because most of the vegetation in our study areas is non-forested, there were very few occasions in which vegetation canopy obscured structures in the imagery. Structures were in all cases at least partly visible, even if they were covered by vegetation, and we looked at earlier images available in Google Earth to confirm where structures were located. The canopy cover was generally lower farther back in time.

Due to the large number of structures in San Diego County, many of which are located in high-density urban core areas, we used a parcel map to facilitate the digitizing process. For small parcels (area <900 m^2^, equivalent to one 30×30 m pixel of the environmental data, see below), we placed the point representing the structure in the centroid of the polygon instead of digitizing the exact location of the structure within the parcel boundary. We assumed the location of the structure within the boundary of small parcels would not significantly alter the overall calculations of spatial pattern among structures. However, for large parcels, the location of the structure within the parcel boundary may be important because the parcel may include more than one pixel, and thus, the environmental data are associated with the structure may depend on structure location. Distance calculations to other structures could also be more substantially influenced by the location of structures in large parcels, which is why we analyzed the Google Earth imagery to place those structures accurately. We did not digitize houses under construction at the date the remote sensing imagery was recorded.

To identify burned structures, we developed an initial address list and spatial database of structures destroyed or damaged by fires from a variety of records, including official incident reports, county assessors' offices, public works departments, city records, and newspaper reports. Because these records were incomplete, we also used Google Earth imagery for a systematic visual analysis to correct geocoded locations and to identify additional structures that had not been documented. For this analysis, we identified burned structures by comparing pre-fire to post-fire images that are available in Google Earth. To develop a data set of houses to inspect for property loss, we selected all structures that fell within and up to 80 m outside any perimeter of a fire that occurred since 2001 in both study areas. We used 80 m because it is twice the distance beyond which flame fronts are not expected to ignite wood [36].The determination of destroyed or damaged structures was based on data collected from official records combined with visual inspection of imagery. Destroyed structures were those in which the house had completely burned to the ground, whereas damaged structures where those that had partially burned. Because damaged structures were more difficult to identify in the imagery, we ruled that if a fire had clearly burned into the property (i.e., if vegetation had visibly been burned), the structure was classified as damaged.

For both the destroyed and damaged structures, we assigned an estimate of certainty for the classification and conducted sensitivity analyses to test if results were similar for destroyed structures that were classified with the highest level of certainty versus a complete dataset with all destroyed and damaged homes at all certainty levels. In our classification, we indicated “1” for uncertain if the house was damaged or destroyed; “2” for fairly certain; “3” for absolutely certain. Since the results were similar (Table 3), we used the full dataset in our analyses to obtain the largest sample size. Although rare, if two buildings burned on a parcel, we only included one in our analysis. For those structures that burned in more than one fire, which only occurred in San Diego, we only used the data for the first fire to avoid double counting of structures in the spatial analysis.

### Explanatory variables

To fully explore the influence of housing arrangement and pattern, we analyzed both the spatial relationships among individual structure locations and the arrangement of structures within housing clusters. Housing clusters were defined as groups of houses with a maximum distance of 100 m from each house to any other house [24]. We calculated these housing clusters by creating a 100 m buffer around each structure and dissolving overlapping boundaries. Thus, areas with many homes within 100 m of each other constituted one large housing cluster, while smaller housing clusters contained fewer or more isolated homes. This allowed spatial analysis based on the spatial and biophysical properties of the structure locations as well as spatial and biophysical properties of the housing clusters within which structures were located. Thus, some variables were calculated for the housing cluster in which the structure was located and the values for that housing cluster were assigned back to the structure. Other variables were calculated only for the location in which the structure was located.

Because our objective was to better understand the landscape factors that significantly contribute to the likelihood that a house will burn in a wildfire, particularly focusing on those factors that are relevant to land use planning, we only assessed variables affecting exposure of structures to wildfires (i.e., fires spreading into the property and reaching the structure, or embers landing on a structure). We did not consider factors such as urban landscaping or housing construction materials within the home ignition zone that determine whether the house survived the exposure. To evaluate the influence of housing arrangement and location on susceptibility to wildfire, we considered a suite of variables representing different spatial configurations and locations of structures as well as additional environmental variables that may affect property loss due to their potential control over fire spread behavior, fuel moisture, or flammability [23], [37] (Table 1).

#### Housing arrangement variables

We evaluated the area of the housing cluster to test the hypothesis that small, isolated groups of structures are more susceptible to wildfire than large groups of structures. Housing density was calculated as the number of structures divided by the area of the housing cluster. For every structure, we calculated the distance to the edge of the housing cluster to evaluate whether structures in the interior of housing clusters were less susceptible to wildfire than structures at the edge. To assess local spatial patterns, we calculated the distance from each structure to its nearest neighbor, and for overall landscape configuration of structures, we calculated the distance from each housing cluster to the next nearest housing cluster. Finally, we calculated the coefficient of variation, or, the standard deviation of distance among structures in a housing cluster divided by the mean to assess housing dispersion, or, regularity of housing pattern.

#### Housing location variables

To test whether structures located in fire-prone parts of the landscape were more likely to be burned, we overlaid fire perimeter polygons compiled by the California Department of Forestry (CDF)-Fire and Resource Assessment Program and created a continuous raster map representing the number of times an area had burned from the beginning of record-keeping, 1878, until 2001. We did not include any fires that occurred after 2001 to ensure that our count of fire frequency was independent of those fires that burned the structures in our analysis. We calculated the distance from the coast for every structure as another way to test whether a structure's location influences its likelihood to be burned. In southern California, a number of variables that influence fire patterns, including climate, terrain, and vegetation distribution, are correlated with the distance to the coast. Distance to the coast is also correlated with housing patterns, and may influence how a house is arranged relative to the major wind corridors in the region [38]. Although the inclusion of weather data at the time of fires would be more directly related to fire behavior and danger, the high variability of weather over space and time limits the ability to relate specific weather data to the place and time that fires burn structures. First, we did not know the exact time that fires burned structures, and thus could not retrieve the temporally matching weather data. Second, weather stations are generally located too far away from where fires burned homes to reflect local variability in weather conditions.

#### Biophysical variables

Terrain-derived variables included the average elevation and percent slope of the housing cluster as well as a cosine-transformation of aspect to create an index of ‘southwestness,’ which could account for the influence of solar radiation and aspect on fuel properties and fire behavior. For each structure, we also determined fuel type in the surrounding by identifying the most common fuel model within a 1 km buffer of the structure. This buffer allowed us to identify the vegetation types fires spread through before reaching the property. Our objective for this analysis was to determine which broad-based fuel classes were most closely associated with structure loss. If more than one fuel type occurred in the buffer, we used the fuel type present in the majority of the area. We obtained spatial fuel model data, developed for fire behavior modeling, from statewide maps developed by the U.S. Forest Service (N. Amboy) at 30 m resolution. The fuel models provided in the USFS maps were created through remote sensing and classified according to Scott and Burgan [39]. From this map, we grouped together the fuel models from broad fuel types (representing grassland, shrubland, and timber). We also grouped agriculture, barren land, and urban land into one type representing mostly urban landscaping and impervious surface (i.e., with little wildland vegetation).

#### Firefighter access

As a way of indirectly assessing firefighter access to the structure, we calculated the length of road within each housing cluster using the 2000 US Topologically Integrated Geographic Encoding and Referencing system TIGER/line files from the US Census.

### Statewide fire hazard maps

Statewide fire hazard maps were available online from the California Department of Forestry and Fire Protection (CDF) [20].We downloaded the Wildland Urban Interface (WUI) “fire threat” data product that includes a series of maps that rank the wildland fire threat to human development. The term “fire threat” in these maps is used analogously to the way we use the term fire “hazard” or, a phenomenon or place where harm is likely to occur.

The “fire threat” map is based on the hazard ranking of different fuels types combined with the fire rotation period, or, the average area burned during the period of record for different vegetation types. Fuels types with higher fuel loads and vegetation types that burned most frequently were considered most hazardous. The “fire threat to people” map is based on a cost-distance calculation that estimates distances from areas of high fire hazard. As an example, the highest “fire threat to people” is calculated as a maximum of 2400 m from “extreme threat” in the fire threat map. Finally, the “communities at risk” map depicts U.S. Census communities with more than 1 house per 8.09 ha (20 acres) that are located in areas with “high fire threat to people.”

The CDF provides additional fire hazard severity maps developed separately for state and local responsibility areas. The finer-scale maps for local responsibility areas, which include incorporated cities, cultivated agricultural lands, and portions of the desert, are limited in extent and only overlap a small portion of our study areas. Due to the limited extent of the local responsibility area maps, and the fact that the state responsibility maps were still being refined, we did include these in our analysis. Their proposed modeling approach will be based upon the existing fire threat and communities at risk maps and will be refined to include additional methods that characterize brand production from vegetative fuels.

To evaluate how well the CDF statewide fire hazard maps corresponded to actual burned structures, we included the three maps as predictor variables in our statistical analyses and quantified the distribution of burned and unaffected structures within the different classes of each map.

### Analysis

To identify the variables that best explain property loss and to estimate the relative contribution of each variable, we developed generalized additive models (GAMs) using a binary response (i.e., house burned or unaffected by fire) and logit link. We used three target degrees of freedom for smoothing splines for our continuous explanatory variables. Because we wanted to compare the independent relative variance explained for all explanatory variables, we estimated separate regression models for each variable. However, we also calculated the correlation coefficients among all variables and developed multiple-regression models with non-correlated variables for each study area. We used a stepwise selection procedure, entering variables according to amount of deviance explained and exploring both forward and backwards directions. We used AIC as the selection criterion for variable selection. To develop the models, we split the data for training and testing (withholding 20% of the data for testing) so we could calculate the area under the curve (AUC) of receiver operating characteristic (ROC) plots on an independent dataset to quantify model performance.

We used GAMs because prior studies reported nonlinear relationships between fire patterns and many of our predictor variables [7], [23], [24]. Unlike parametric statistical methods, such as generalized linear models (GLMs), in which nonlinear relationships are specified *a priori* (e.g., through polynomial terms) in the model, GAMs allow the structure of the data to determine the shape of the response curves. Thus, GAMs provide a more flexible and automated approach for identifying and describing nonlinear relationships [40], [41]. We used the GAMs to estimate the shape of response curves and to calculate deviance explained (D^2^, analogous to R-squared in linear regression) for all explanatory variables.

Although non-parametric methods, such as GAMs, tend to be less sensitive to the effects of spatial autocorrelation than other model approaches [42], we wanted to ensure that spatial autocorrelation did not significantly influence the results of our analysis. The main concerns about spatial autocorrelation in regression models are inflated significance values and biased coefficients [42], [43]. GAMs do not estimate regression coefficients, which are replaced with smoothing functions. This is why we also fit GLMs to our data because they are parametric models similar to GAMs, but they estimate coefficients. Therefore, the GLMs allowed us tocheck the influence of autocorrelation on both coefficients and the significance of variables. The GLMs also allowed us to test whether our results were robust by comparing two modeling methods. We first developed non-spatial GLMs, and fit linear and quadratic terms for all variables (except for fuel type, which was categorical). After detecting residual autocorrelation in these nonspatial models using Moran's I [43], we calculated an autocovariate term to account for the influence of neighboring values on predictions, and included as the term as an additional explanatory variable in models. To calculate the autocovariate term, we specified a neighborhood radius of 1, which finds the minimum distance for which all observations (i.e., structure locations) are linked to at least one neighbor. The influence of structures located within any neighborhood radius was weighted by inverse distance. . After fitting these autocovariate models, we used Moran's I to recheck for spatial autocorrelation of model residuals, compared the coefficients to the nonspatial models, and checked variable significance after incorporating the autocovariate term..All model fitting and evaluation were accomplished using the gam, spdep, vegan, and ROCR packages for R [44].

### Empirical fire mapping

To develop empirical fire hazard models and maps, we selected Maxent [45], a machine-learning method that is best recognized for creating species distribution models and maps. We selected Maxent because it outperforms other presence-only and presence-background species distribution modeling methods [41] and has been applied successfully to map the distribution of fire [46]. Maxent assumes that the best approximation of an unknown distribution (e.g., fire hazard) is the one with maximum entropy. The model iteratively evaluates contrasts between values of explanatory variables at locations of the response variable (i.e., burned structures) and for averages of the explanatory variables across the entire study area. The output is an exponential function that assigns a hazard probability (i.e., probability of structure being burned) to each site or cell of a map. In the output map, areas of predicted high risk that do not have structures on them represent environmental conditions similar to those in which structures have actually burned.

Because mapped predictor variables were required for the modeling, so that conditions similar to those where structures were burned could be delineated continuously across the landscape, we created maps representing a subset of the variables that we explored with the regression analysis. These variables represented a combination of structure arrangement, location, and biophysical variables, including: interpolated structure density, distance to coast, fuel type, slope, historical fire frequency, and southwestness. We developed models that included CDF fire hazard maps as predictors to test their importance relative to the other predictor variables. However, for generating maps and quantifying model performance, we only used models that did not include CDF predictor variables.

### Sensitivity tests

The results of our analysis may have been affected by the size of the buffer that we used around structures to create housing clusters, the degree of impact of fire on the structure (i.e., destroyed or damaged), and certainty of the classification (i.e., 1–3). Therefore, to evaluate how sensitive our results were to these variables, we created housing clusters around structures using a 200 m buffer and compared the regression results for which housing cluster was relevant in the to those obtained when using a 100 m buffer. We also performed separate regressions using only those structures that had been destroyed with complete certainty (a “3”) and compared those to the regressions of all burned structures at all certainty levels. For the Maxent analysis, we also compared models using only structures that were destroyed with the highest level of certainty to models using all burned structures at all certainty levels.
